# Proteome Informatics in Tibetan Sheep (*Ovis aries*) Testes Suggest the Crucial Proteins Related to Development and Functionality

**DOI:** 10.3389/fvets.2022.923789

**Published:** 2022-07-15

**Authors:** Taotao Li, Huihui Wang, Ruirui Luo, Xuejiao An, Qiao Li, Manchun Su, Huibin Shi, Haolin Chen, Yong Zhang, Youji Ma

**Affiliations:** ^1^College of Animal Science and Technology, Gansu Agricultural University, Lanzhou, China; ^2^Gansu Key Laboratory of Animal Generational Physiology and Reproductive Regulation, Lanzhou, China; ^3^Animal Husbandry, Pasture, and Green Agriculture Institute, Gansu Academy of Agricultural Sciences, Lanzhou, China; ^4^Guizhou Institute of Animal Husbandry and Veterinary Science, Guiyang, China

**Keywords:** sheep, testis, spermatogenesis, germ cell, Sertoli cell, data-independent acquisition, targeted proteomics

## Abstract

Testis has an indispensable function in male reproduction of domestic animals. Tibetan sheep (*Ovis aries*) is a locally adapted breed of sheep raised in the Qinghai-Tibet Plateau, with outsized roles in providing the livelihood for millions of residents. Nevertheless, less is known on how protein expression and their functional roles in developmental testes of such breed limit their use in breeding efforts. In this study, we obtained comprehensive protein profiles from testes of Tibetan sheep at three developmental stages (including pre-puberty, post-puberty, and adulthood) using data-independent acquisition-based proteomic strategy to quantitatively identify the differentially abundant proteins (DAPs) associated with testicular development and function and to unravel the molecular basis of spermatogenesis. A total of 6,221 proteins were differentially expressed in an age-dependent manner. The reliability of the gene expression abundance was corroborated by quantitative PCR and targeted parallel reaction monitoring. These DAPs were significantly enriched to biological processes concerning spermatid development and sperm deformation, mitosis, glycolytic process, cell-cell/extracellular matrix (ECM) junctions, cell proliferation, apoptosis, and migration and to the pathways including, developmental process and sexual reproduction-related (such as VEGF, estrogen, insulin, GnRH, Hippo, PI3K-Akt, mTOR, MAPK, and AMPK), and testicular cell events-related pathways (such as tight/gap/adherens junctions, ECM-receptor interaction, regulation of actin cytoskeleton, glycolysis, cell cycle, and meiosis). Based on these bioinformatics analysis, we constructed four protein–protein interaction network, among which the proteins are involved in mitosis, meiosis, spermiogenesis, and testicular microenvironment, respectively. Altogether, these bioinformatics-based sequencing results suggest that many protein-coding genes were expressed in a development-dependent manner in Tibetan sheep testes to contribute to the testicular cell development and their surrounding microenvironment remodeling at various stages of spermatogenesis. These findings have important implications for further understanding of the mechanisms underlying spermatogenesis in sheep and even other plateau-adapted animals.

## Introduction

In males, testis is a crucial reproductive organ responsible for spermatogenesis and androgen secretion. Testicular organogenesis is a complicated, continual, exquisite, and multilevel physiological process that involves morphological and functional changes in different testicular compartments (1, 2). The testicular development is predominantly characterized by the development and differentiation of germ cells (GCs), as well as somatic cells, such as Sertoli cells (SCs) (3, 4). It has been known that spermatozoa develop from undifferentiated spermatogonia in mammalian testis through the process of spermatogenesis, involving spermatogonial mitotic proliferation, two successive meiotic divisions, and dramatic morphological changes from haploid spermatids to mature sperm through spermiogenesis (including nuclear condensation, acrosome formation, and assembly of flagellum). Beyond this, somatic cells constitute the testicular microenvironment or niche, which is essential for continuous spermatogenesis (5). For instance, SCs are essential for spermatogenesis not only through directly interacting with GCs within the seminiferous epithelium but also by forming the blood–testis barrier (BTB) to provide morphological, nutritional, and immune support for GC development; their dysfunction is often the cause of spermatogenic failure (6). Spermatogenesis is modulated *via* intimate interactions between somatic and GCs, which requires coordinate changes in gene and protein expression (7–9).

Previous studies with mammalian testis toward understanding gene expression and signaling events have primarily evaluated processes at the transcriptional level (10–12), and the same is true for sheep (13, 14). Nevertheless, the transcriptional regulation is not enough to reflect the level of gene expression and its duration because gene expression may be extensively regulated at both the transcriptional and posttranscriptional levels, and the current proteomics-based investigations of expression and regulation of the functional genes within mammalian testis remain very limited (7). In domestic animals, while the proteomic technology is now widely used in the developmental ovine (15), bovine (16), and porcine (17) testes, the conventional and low-throughput proteomic methods, such as two-dimensional gel electrophoresis, were mostly adopted, which limited our understanding of testicular proteome. In contrast to these conventional proteomic techniques and reporter-ion-based proteomic quantification methods (e.g., tandem mass tagging and isobaric tags for relative and absolute quantitation), the data-independent acquisition (DIA)-based quantitative proteomic strategy, a recently developed technology, presents unique advantages, such as broad proteomic coverage, good reproducibility, and high quantitative accuracy (18). This high-throughput quantitative strategy provides the opportunity for the in-depth mining of its gene space and greatly advance our comprehensive understanding of the molecular mechanisms of gene expression regulation in response to testis development and spermatogenesis in sheep.

Tibetan sheep is an aboriginal breed in the Qinghai-Tibetan Plateau area at altitudes over 3,000 m characterized by late sexual maturity, low reproductive rates, and high resistance to high-altitude hypoxic environment. Therefore, it has considered as excellent models to investigate reproductive performance of sheep including GC development and testis function. Protein fluctuations are essential for biological function, and thus, elucidating the proteome in Tibetan sheep testis is important for understanding the functional roles of proteins in regulating spermatogenesis and testicular development in the ovine species.

## Materials and Methods

### Animals and Samplings

For this investigation, we used 24 healthy, purebred, male Tibetan sheep in total (location Xiahe, China; altitude > 3,000 m): eight from 3-month-old (pre-puberty), eight from 1-year-old (post-puberty), and eight from 3-year-old (adult). All right testicular tissues mentioned above were quickly dissected, rinsed with PBS, flash-frozen in liquid nitrogen, and stored at −80°C.

### Protein Isolation and Digestion

The frozen testicular samples were dissolved in lysis buffer (2% sodium dodecyl sulfate, 7M urea, 1 mg/ml protease inhibitor cocktail) and lysed by sonication on ice three times for 180 s each. The homogenate was centrifuged at 4°C, 14,000 rpm for 30 min to acquire the supernatant. A Pierce BCA Protein Assay Kit (Thermo Scientific, Rockford, USA) was used to quantify the total protein in each supernatant. The supernatant lysates containing 50 μg of protein were diluted to a total volume of 50 μl and then added with 1 μl of DTT (1 M) for incubation at 55°C for 1 h. After this, 5 μl of iodoacetamide (1 M) was added and incubation continued for 1 h, protected from light, at 37°C, and then mixed with 300 μl of cold acetone and settled for 2 h at −20°C, followed by digestion overnight with 20 μg of trypsin (Promega, Madison, WI, USA) per mg protein.

### DIA-Based Proteomic Analysis

#### High pH Reversed-Phase Separation

The peptide mixture was redissolved in the buffer A (20 mM ammonium formate in water, adjusted pH to 10.0 with ammonium hydroxide) and then fractionated by high pH separation using Ultimate 3000 system (ThermoFisher scientific, Waltham, MA, USA) connected to a XBridge C18 reverse-phase column (4.6 × 250 mm, 5 μm; Waters, Milford, MA, USA). High pH separation was performed using a linear gradient, starting from 5% B to 45% B in 40 min (B: 20 mM ammonium formate in 80% acetonitrile, adjusted pH to 10.0 with ammonium hydroxide). The column was re-equilibrated at the initial condition for 15 min. The column flow rate was maintained at 1 ml/min, and the column temperature was maintained at 30°C. A total of ten fractions were collected and dried in a vacuum concentrator for later use.

#### Mass Spectrometry: Data-Dependent Acquisition

The desalted and lyophilized peptides were mixed with 30 μl of 0.1% formic acid in water and analyzed with on-line nanospray liquid chromatography with tandem mass spectrometry (LC-MS/MS) on an Orbitrap Fusion Lumos coupled to EASY-nLC 1,200 system (Thermo Fisher Scientific, Waltham, MA, USA). A volume of 3 μl of peptide sample was loaded onto the analytical column (Acclaim PepMap C18, 75 μm × 25 cm) and separated with a 120 min gradient, from 5 to 35% C (C: 0.1% formic acid in acetonitrile). The column flow rate was held at 200 nl/min at 40°C, with an electrospray voltage of 2 kV. The mass spectrometer was run under data-dependent acquisition mode with automatic switching between MS and MS/MS acquisition. The mass spectrum parameter settings were as follows: (1) MS: scan range = 350–1,500 m/z; resolution = 120,000; automatic gain control (AGC) target = 4e5; maximum injection time = 50 ms; dynamic exclusion time = 30 s and (2) higher energy collisional dissociation (HCD)-MS/MS: resolution = 15,000; AGC target = 5e4; maximum injection time = 35 ms; collision energy = 32.

#### Database Searching of DDA Data

Raw data of DDA were analyzed using the Spectronaut X software (Biognosys AG, Switzerland) with default settings to generate an initial target list. Spectronaut was set up to search the database of *Ovis aries* along with contaminant database assuming trypsin as the digestion enzyme. Carbamidomethyl (C) and methionine (M) oxidation were specified as the fixed and variable modifications, respectively. We applied a false discovery rate (FDR) value of 1% to filter the search results on peptide precursor and protein level.

#### Separation Mass Spectrometry: DIA

The peptides were resuspended into 30 μl of 0.1% formic acid; 9 μl was harvested and mixed with 1 μl of 1× iRT standard, and then analyzed by online electrospray MS/MS. The MS was run under data-independent acquisition program and automatically switched between MS and MS/MS mode. The settings were: (1) MS: scan range = 350–1,500 m/z; resolution = 120,000; AGC target = 4e6; maximum injection time = 50 ms and (2) HCD-MS/MS: resolution = 30,000; AGC target = 1e6; collision energy = 32 with 5% stepped collision energy. (3) DIA data were collected with 60 variable isolation windows, and each window overlapped 1 m/z.

#### Protein Identification and Quantitation

DIA raw data were analyzed using Spectronaut X (Biognosys AG, Switzerland) with a default setting. Concisely, the retention time prediction type was set to dynamic iRT, and the correction factor for the window was set to one. Data extraction was carried out by Spectronaut X based on the extensive mass calibration. Interference correction was on the MS2 level. The FDR was set to a maximum of 1% at peptide precursor level to allow positive protein identifications. The protein intensity was summed by the intensity of their respective peptides, which were calculated according to the peak areas of their respective fragment ions of MS2.

#### Protein Identification and Quantitation

The proteins with the absolute value of fold change > 1.5 and *q*-value < 0.05 were designated as differentially abundant proteins (DAPs). The clustering analysis of temporal profiles of gene expression was carried out using the R MFuzz package using the fuzzy c-means clustering algorithm (19). The DAVID database (https://david.ncifcrf.gov/summary.jsp) and KOBAS database (http://kobas.cbi.pku.edu.cn/annotate/) were applied to infer their potential functions in gene ontology (GO) and Kyoto Encyclopedia of Genes and Genomes (KEGG) annotations. Significant GO functions and pathways were measured within DAPs with *p-*value ≤ 0.05. The protein–protein interaction (PPI) network of DAPs was constructed using STRING online database, followed by visualization by Cytoscape 3.7.0.

### qPCR Verification

The expression of 18 DAPs identified by the MS assay was assessed by quantitative PCR (qPCR). Briefly, testicular tissues were lysed in Trizol (TransGen, Beijing, China) to harvest total RNA. cDNA was prepared by amplifying 500 ng of RNA by Evo M-MLV RT Kit (Accurate Biotechnology, Hunan, China). qPCR detection of mRNA expression relative to β-actin was carried out using SYBR Green qPCR Kit (Accurate Biotechnology, Hunan, China). Eight independent biological replicates were included in the qPCR analysis. Specific primer sequences are listed in the Supplemental Table S1. The data were expressed as relative quantification calculated as 2^−ΔΔCt^.

### Quantitative Validation Based on Parallel Reaction Monitoring Targeted Proteomics

DAPs were chosen for validating discovery DIA proteome data through targeted parallel reaction monitoring (PRM) mass spectrometry method on the identical LC-MS system as above but adjustment for PRM setting. In brief, the equal amount peptides from each sample were redissolved in 0.1% formic acid in water and analyzed by on-line nanospray LC-MS/MS on Orbitrap Fusion Lumos mass spectrometer (Thermo Fisher Scientific, MA, USA) coupled to an EASY-nanoLC 1200 system (Thermo Fisher Scientific, MA, USA). A volume of 1.5 μl peptide was loaded (analytical column: Acclaim PepMap C18, 75 μm × 25 cm) and separated with a 120 min gradient. The column flow rate was maintained at 300 nl/min with the column temperature of 40°C. The electrospray voltage of 2 kV vs. the inlet of the mass spectrometer was used. PRM settings were as follow: Full MS scans in the mass range from m/z 350 to 2,000 were acquired with a resolution of 120,000. MS2 spectra were acquired with a resolution of 30,000, and the maximum ion injection time and the AGC target were 54 ms and 5e4, respectively. The inclusion list exported from SpectroDive was imported into the mass list table in the PRM mode. Raw files of the targeting runs were analyzed using SpectroDive 9.10 with the default settings. Q-value cutoff on precursor was ≤ 1%. The average of filtered peptides was used to calculate the protein quantities.

### Statistical Analysis

Multiple comparisons were carried out using one-way ANOVA followed by the Turkey multiple comparison test. Error bar shows ± SD. *p*-values of < 0.05 and < 0.01 were considered significant and extremely significant, respectively.

## Results

### Analysis of Differentially Abundant Proteins

**Figure 1:**
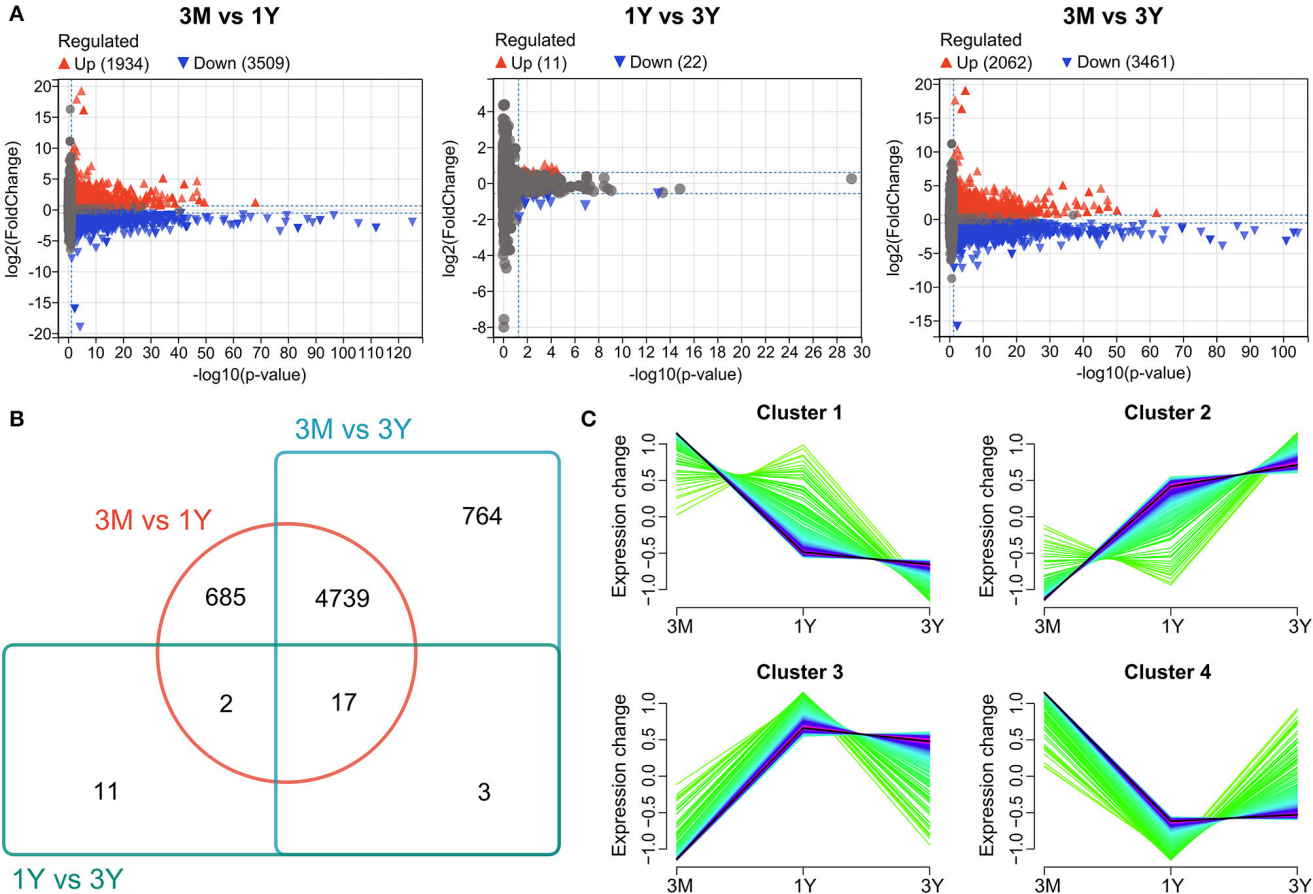
Differential protein expression profiles. **(A)** Volcano plot showing protein expression profiles between 3M and 1Y groups, 1Y and 3Y groups, and 3M and 3Y groups. **(B)** Venn diagram showing the shared and unique proteins. **(C)** Cluster analysis of proteins showing similar pattern of expression during testis development. 3M, 1Y, and 3Y correspond to 3-month-old, 1-year-old, and 3-year-old, respectively.

Differential expression analysis discovered that in total, 5,443 proteins (among a total of 12677 identified proteins) were significantly differentially expressed between 3-month-old and 1-year-old (Figure 1A), of which 1,934 ones were upregulated and 3,509 ones were downregulated in 1-year-old group; 33 proteins (among a total of 13,715 identified proteins) were significantly differentially expressed between 1-year-old and 3-year-old (Figure 1A), of which 11 ones were upregulated and 22 ones were downregulated in 3-year-old group. Among these, 17 DAPs were co-expressed in testes from three age groups; 685, 764, and 11 DAPs were expressed independently in 3M vs. 1Y, 3M vs. 3Y, and 1Y vs. 3Y, respectively; 4,756, 19, and 20 DAPs were co-expressed in 3M vs. 1Y and 3M vs. 3Y, 3M vs. 1Y and 1Y vs. 3Y, 3M vs. 3Y and 1Y vs. 3Y, respectively (Figure 1B). All of DAPs were used for cluster analysis. The results showed that cluster 1 had 818 proteins correspond to 454 genes, and the expression levels of these proteins exhibited a generally declining profile (Figure 1C). Cluster 2 had 2,303 proteins correspond to 1,323 genes, and the expression levels of these proteins presented an overall increasing trend (Figure 1C). Cluster 3 had 717 proteins corresponding to 409 genes, and the expression levels of these proteins showed a first rising and then decline trend (Figure 1C). Cluster 4 had 2,383 proteins correspond to 1,296 genes, and their expression levels exhibited first a decreasing trend and then increase with age (Figure 1C).

### Functional Annotation of Differentially Abundant Proteins

**Figure 2:**
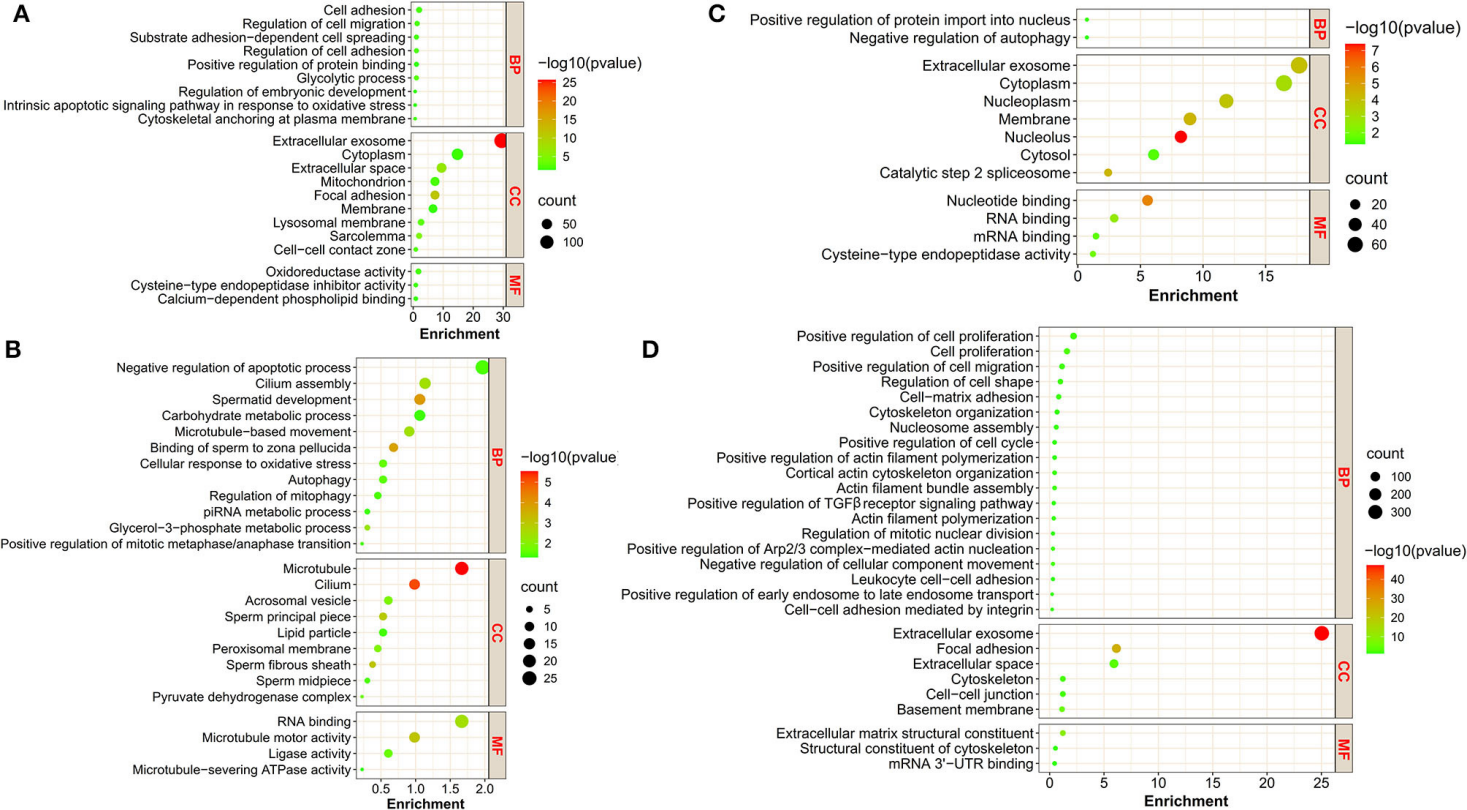
The GO annotation of DAPs in the four clusters. **(A–D)** represent the bubble plots of the significant GO terms for DAPs in cluster 1, cluster 2, cluster 3, and cluster 4, respectively. BP, biological process; CC, cellular component; MF, molecular function.

To associate the changes of protein expression with biological functions, we classified these proteins in clusters using the GO program. The proteins in cluster 1 were significantly enriched for GO terms, including cell–cell (intercellular) adhesion, glycolytic process, and regulation of embryonic development (Figure 2A); the proteins in cluster 2 were mainly implicated in GO terms, including spermatid development and sperm deformation (such as cilium assembly, microtubule-based movement, sperm fibrous sheath and principal piece), positive regulation of mitosis, and negative regulation of apoptotic process (Figure 2B); the proteins in cluster 3 were enriched with GO terms, including positive regulation of protein import into nucleus, negative regulation of autophagy, and RNA binding (Figure 2C); the proteins in cluster 4 were involved in a variety of cellular processes, including cell proliferation, migration, cell–cell and/or cell–matrix adhesion, intercellular junction assembly, and actin skeleton regulation (Figure 2D). Details can be found in Supplementary Tables S2–S5.

**Figure 3:**
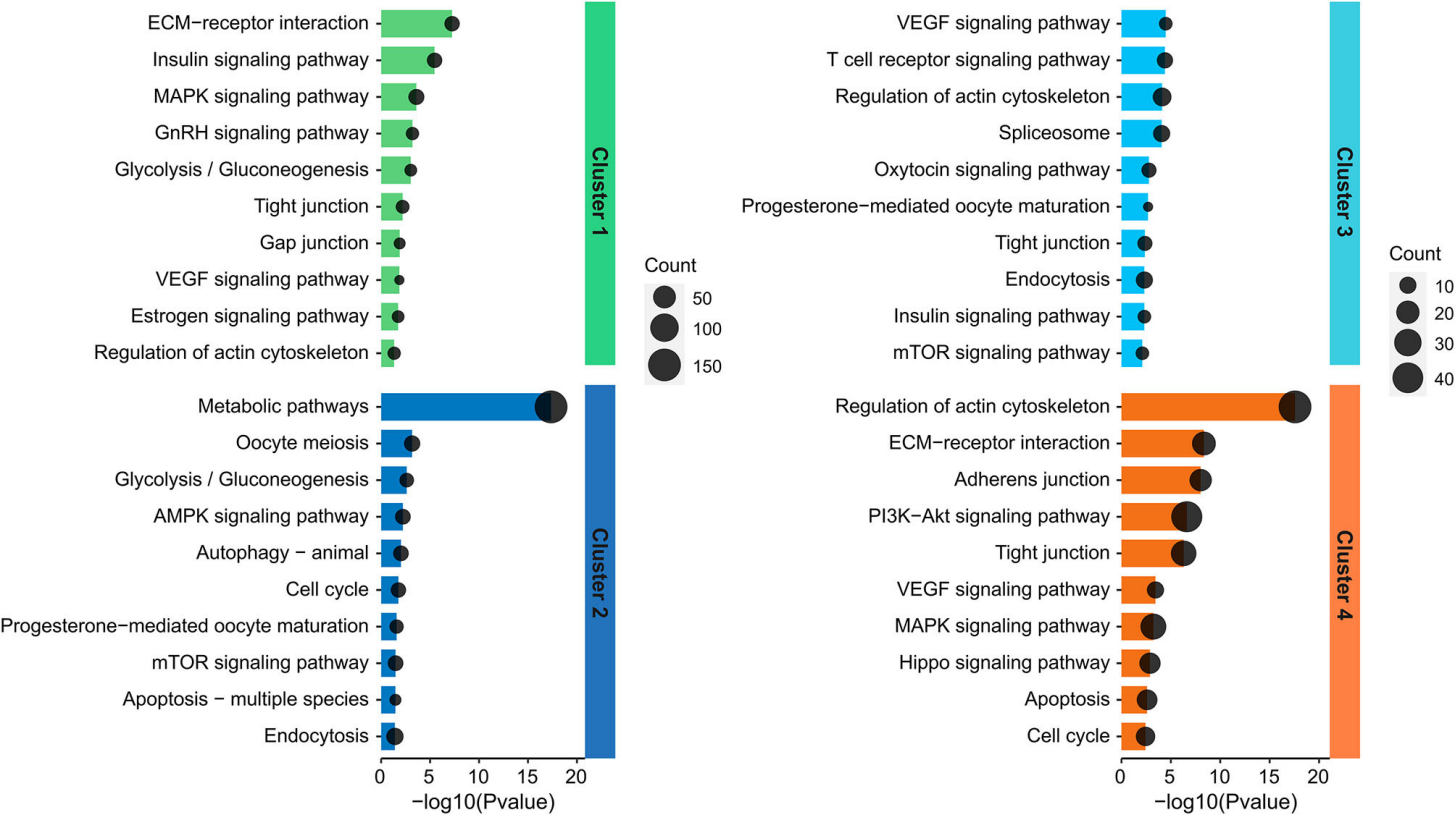
Representative significantly enriched KEGG pathway categories of DAPs in the four clusters.

To better understand the functions played by the identified proteins, KEGG analysis was conducted for identification of associated biologic pathways (Figure 3). From the analysis of Figure 3, the proteins in cluster 1 were significantly involved in the pathways associated with reproduction (such as VEGF, estrogen, insulin, and GnRH signaling pathway) and testicular cell development (such as ECM-receptor interaction, tight junction, gap junction, MAPK signaling pathway, and glycolysis/gluconeogenesis); the proteins in cluster 2 were mostly engaged in pathways associated with reproduction (such as progesterone-mediated oocyte maturation, oocyte meiosis, mTOR, and AMPK signaling pathway), testicular cell events (such as cell cycle, apoptosis, autophagy, and endocytosis), and metabolism including glycolysis; the proteins in cluster 3 were mainly participated in pathways related to reproduction (e.g., progesterone-mediated oocyte maturation, VEGF, mTOR, oxytocin, and insulin signaling pathway), testicular cell development (e.g., tight junction, regulation of actin cytoskeleton, and endocytosis); the proteins in cluster 4 were mainly implicated in spermatogenesis-related pathways, including VEGF, Hippo, PI3K-Akt, cell cycle, apoptosis, regulation of actin cytoskeleton, ECM-receptor interaction, and tight/adherens junction (Figure 3). For detailed data, refer to Supplementary Tables S6–S9.

### Validation of DAPs by PRM Analysis

**Figure 4:**
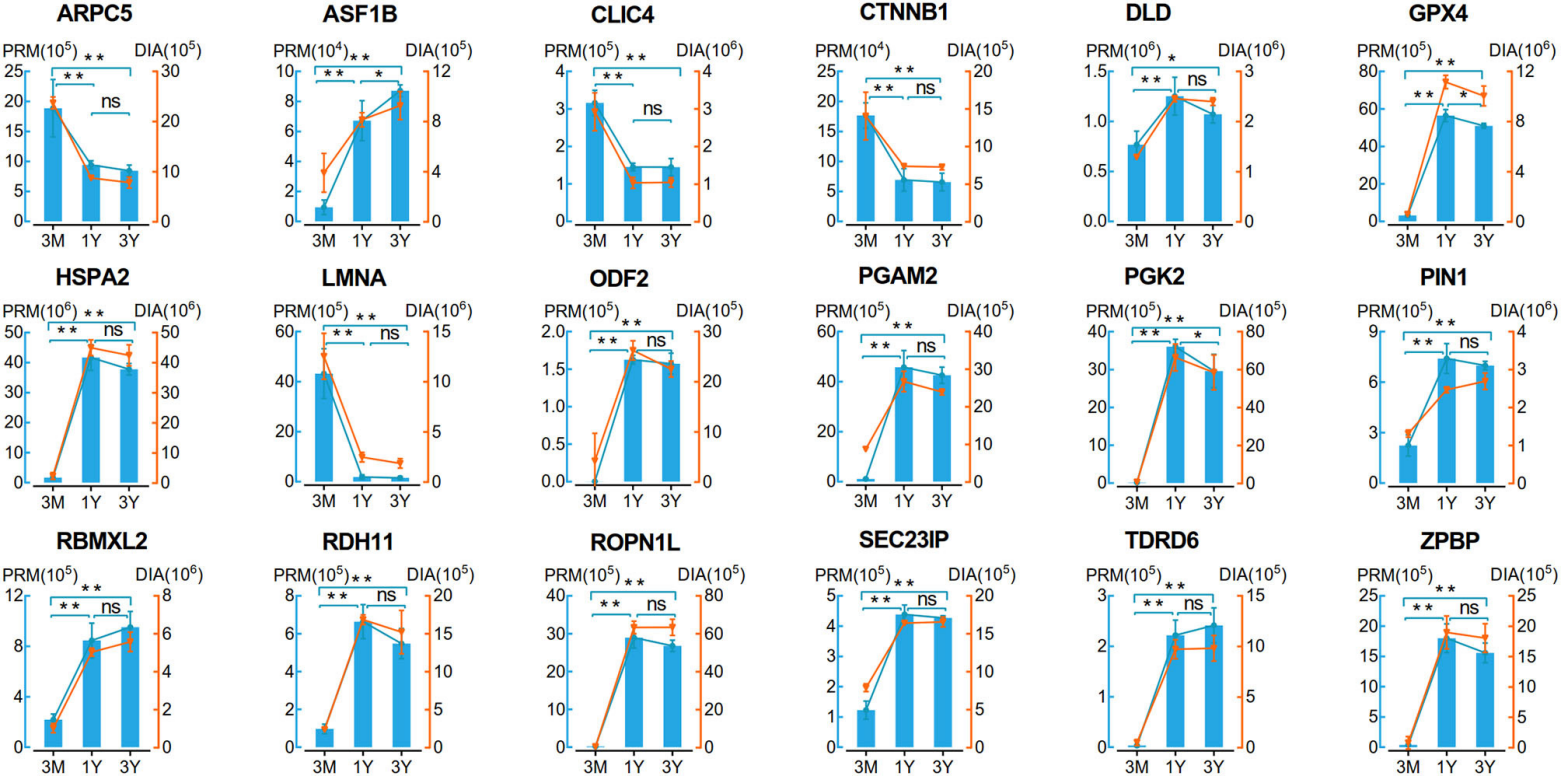
Verification of 18 proteins with differential abundance identified by DIA analysis by PRM (*n* = 4 per group). Data represent the mean ± SD. Significance levels: two asterisks, *p*, 0.01; one asterisk, *p*, 0.05; ns, not significant. 3M, 1Y, and 3Y correspond to 3-month-old, 1-year-old, and 3-year-old, respectively.

To validate the reliability of DIA data, we selected 18 DAPs enriched in the potential biological processes or/and pathways associated with testicular function for the PRM assay. As shown in Figure 4, the protein expression patterns obtained by PRM were in agreement with the expression pattern inferred by DIA data. Of these proteins, 11 proteins (i.e., DLD, GPX4, HSPA2, ODF2, PGAM2, PGK2, PIN1, RDH11, ROPN1L, SEC23IP, and ZPBP) showed a trend of increasing first and then decreasing, four proteins (i.e., ARPC5, CLIC4, CTNNB1, and LMNA) showed a clear pattern of decreasing expression, and the remaining three proteins (i.e., ASF1B, RBMXL2, and TDRD6) were upregulated (Figure 4). These demonstrated the high reliability and credibility of the DIA data in proteomic analysis.

### Validation of DAPs by qPCR Analysis

**Figure 5:**
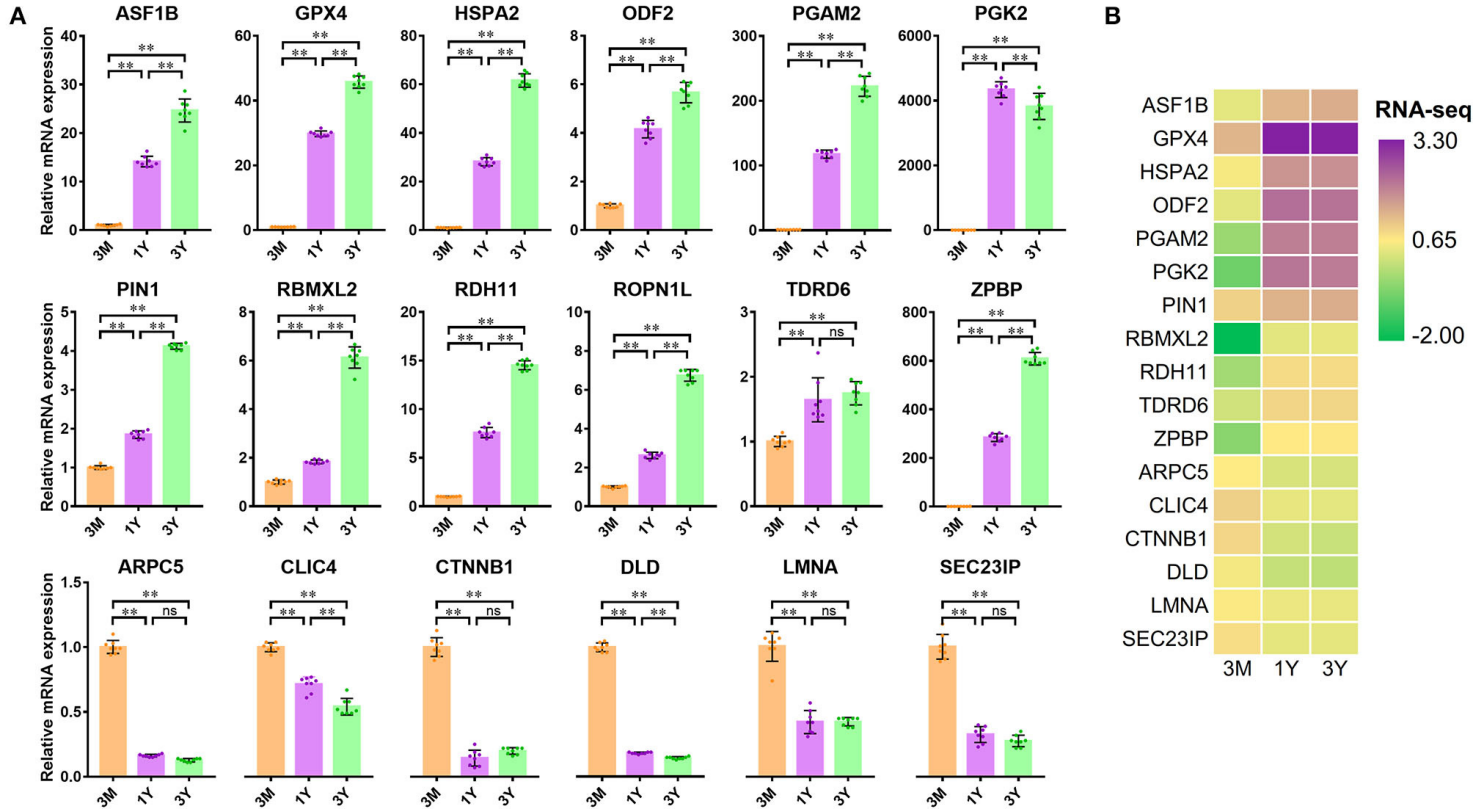
Verification of 18 differentially abundant proteins at the mRNA level (*n* = 8 for each group). **(A)** Relative mRNA expression monitored by qPCR. **(B)** Heatmap of mRNA expression derived from our RNA-seq data (NCBI SRA accessions: SRR11348536–SRR11348547). Significance levels: two asterisks, *p*, 0.01; ns, not significant. 3M, 1Y, and 3Y correspond to 3-month-old, 1-year-old, and 3-year-old, respectively.

We also investigated the mRNA expression levels of these DAPs used for PRM validation by qPCR. In contrast to 3-month-old group, twelve mRNAs corresponding to the proteins showed a clear pattern of increasing gene expression, while six mRNAs show the opposite trend in 1-year-old and 3-year-old groups (Figure 5A). The relevant mRNA expression revealed by qPCR was confirmed in our previous RNA-seq data (Figure 5B). Among these genes, the expression of 16 ones at the mRNA level showed similar trends with protein expression, while the expression level of two genes (i.e., DLD and SEC23IP) appeared largely different and even opposite. This may have been due to the translation regulation between mRNA and protein.

### Protein-Coding Genes Associated With Spermatogenesis

**Figure 6:**
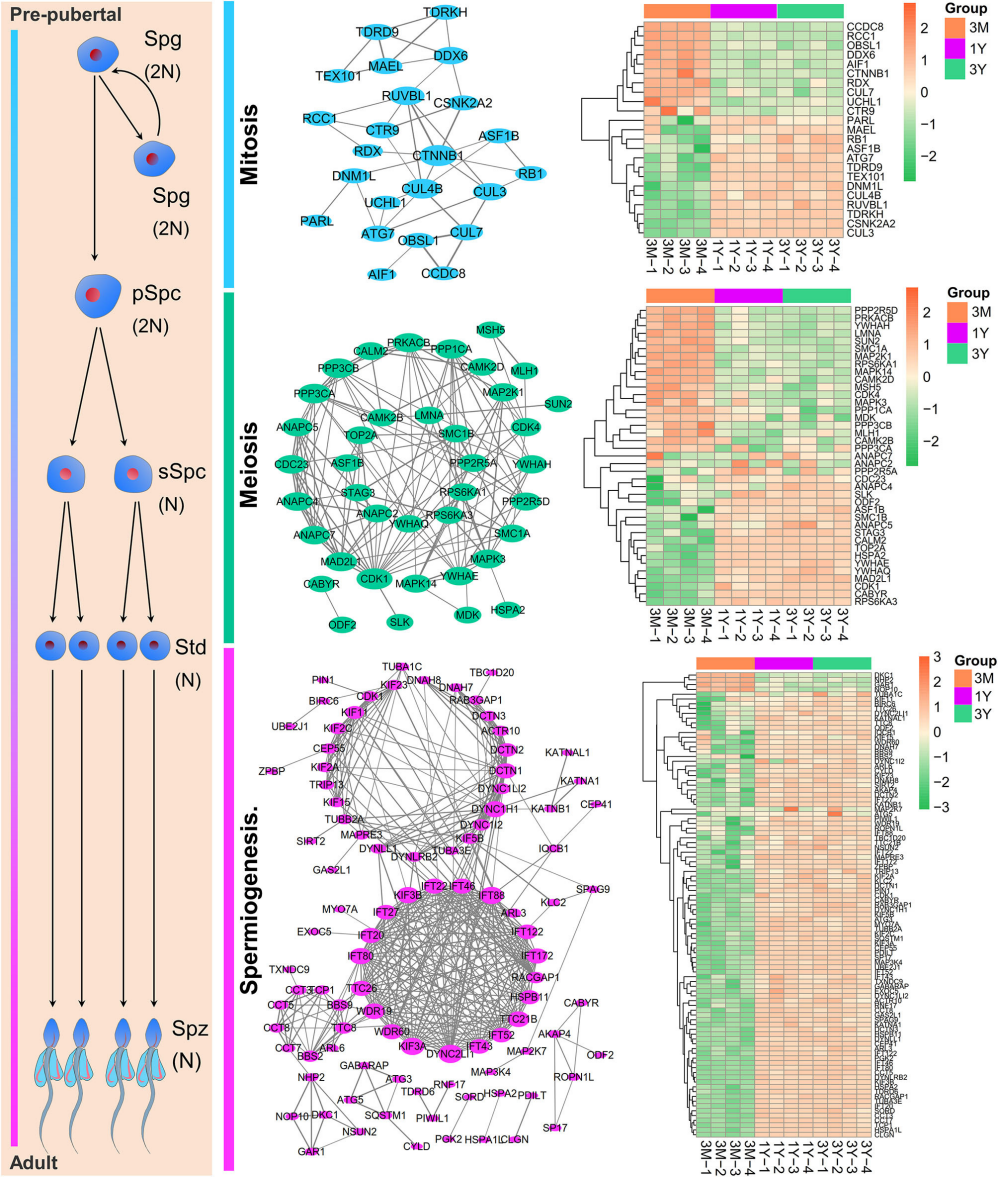
The PPI network and expression heat map of DAPs enriched at different stages of spermatogenesis. Node size indicates surrounding gene numbers (degrees) in the network. Spg, spermatogonia; pSpc, primary spermatocyte; sSpc, secondary spermatocyte; Std, spermatid; Spz, spermatozoa. 3M, 1Y, and 3Y correspond to 3-month-old, 1-year-old, and 3-year-old, respectively.

By integrating the database-based and literature-based functional annotations, we identified several protein-coding gene sets associated with the process of spermatogenesis. Among these, 23 genes presented in PPI network were annotated as functions related to mitosis (mitotic renewal of spermatogonia), which included 13 upregulated and 10 downregulated proteins in postpubertal and adult testes compared with the prepuberty, three (i.e., CTNNB1, CUL4B, and RUVBL1; degree > 5) of which were interacted the top three most with other genes; 39 genes presented in PPI network were annotated as functions related to meiosis (meiotic division of spermatocytes), which included 19 upregulated and 20 downregulated proteins in postpubertal and adult testes compared with the prepuberty, three (i.e., CDK1, PPP3CA, and PPP3CB; degree ≥ 13) of which were interacted the top three most with other genes; 97 genes presented in PPI network were annotated as functions related to spermiogenesis (morphological differentiation of haploid spermatids), which included 93 upregulated and 4 downregulated proteins both in postpubertal and adult testes compared with the prepuberty, three (i.e., DYNC2LI1, KIF3A, and IFT88; degree > 20) of which were interacted the top three most with other genes (Figure 6).

### Identification of Protein-Coding Genes Associated With Testicular Microenvironment

**Figure 7:**
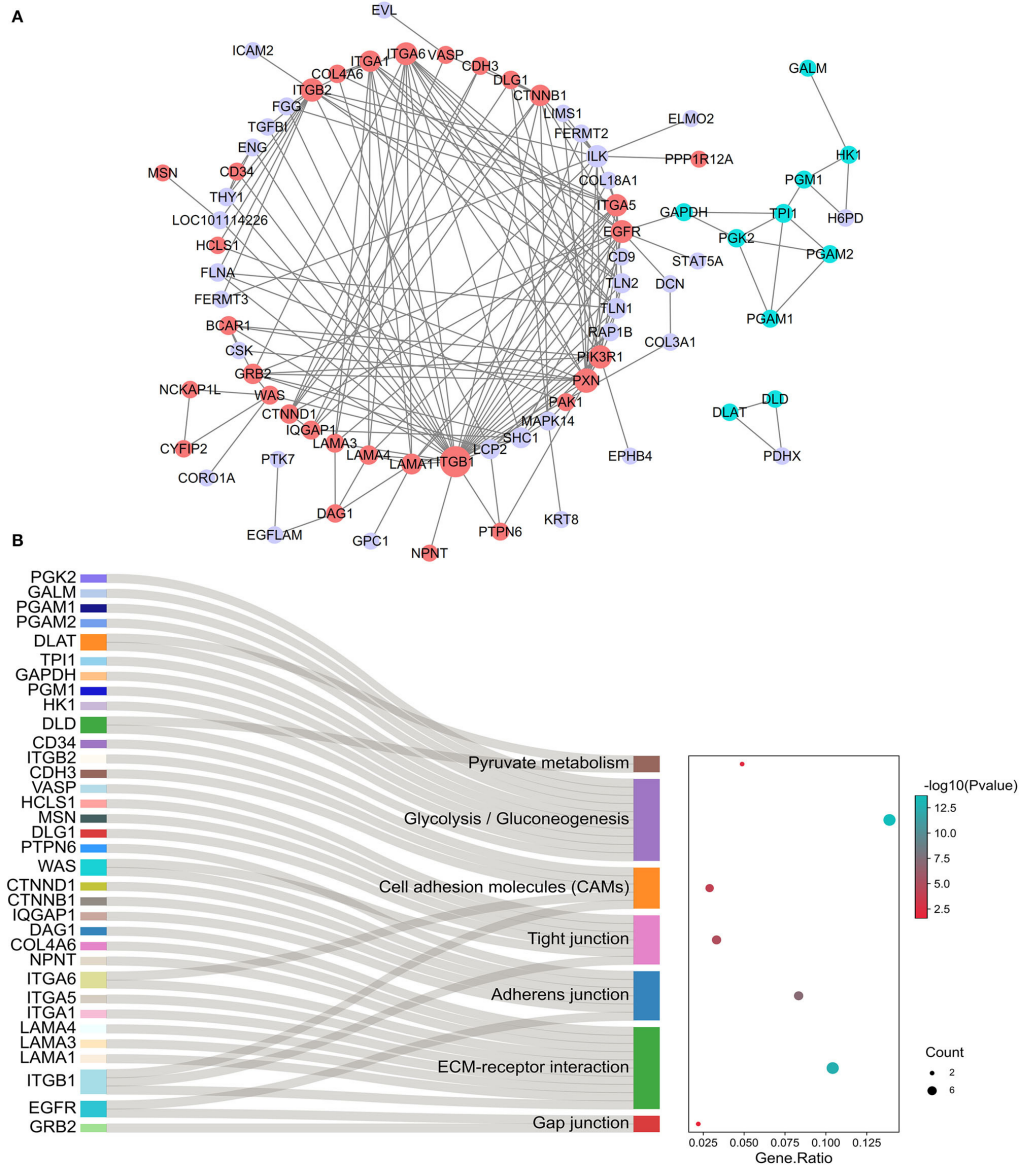
Identification of testicular microenvironment-relevant proteins. **(A)** The PPI network of protein-coding gene sets enriched on testicular microenvironment-related pathways. Network nodes represent proteins, and network edges represent protein-protein interactions. Node size indicates surrounding protein numbers (degrees) in the network. The proteins correlated with cell junctions are shown in red, and proteins correlated with glycolysis/gluconeogenesis are shown in blue. **(B)** The gene-pathway network according to cell junctions and glycolysis-related genes and their enriched pathways.

The PPI network of genes closely associated with testicular microenvironment, with 74 nodes and 175 edges, is presented in Figure 7A. The top 10 hub genes (degree ≥ 10) in the network were integrin proteins (i.e., ITGA1, ITGA5, ITGA6, ITGB1, and ITGB2), CTNNB1, ILK, EGFR, PIK3R1, and PXN. Among them, most of proteins were mainly involved in cell-cell junctions, cell motility, and nutritional support of spermatogenesis. Specifically, 16 genes were involved in regulation of actin cytoskeleton, 10 genes were involved in ECM–receptor interaction, 6 genes were involved in adherens junction, 6 genes were involved in tight junction, 5 genes were involved in cell adhesion molecules, 2 genes were involved in gap junction, and 10 genes were involved in glycolysis and pyruvate metabolism (Figure 7B).

## Discussion

The testis development is mainly characterized by the development and differentiation of GCs and SCs within the seminiferous epithelium. Accumulating evidence shows that this process requires the sequential and coordinated expression of a series of protein-coding genes, including many testis- or male GC-specific gene products (8, 20, 21). The development and differentiation of male GCs are highly orchestrated dynamic processes, which encompass mitosis, meiosis, and spermiogenesis. During mammalian spermatogenesis, expression of most genes is stage-dependent (22, 23). To understand the functions of genes in mammalian testis, investigation of the gene product, the protein, is inescapable. In this study, a total of 6,221 DAPs were identified in Tibetan sheep testes among three developmental stages (pre-pubertal, post-pubertal, and adult), exhibiting four developmental phase-dependent expression patterns. The subsequent results generated by PRM and qPCR analysis confirmed the reliability of DIA data analysis. Notably, not all of the genes were altered synchronously at mRNA and protein level at different stages of testis development, such as DLD, GPX4, HSPA2, and SEC23IP. This might be due to either high mRNA turnover or high protein stability, as well as frequent posttranscriptional regulation during mammalian spermatogenesis (24).

The proteins with generally decreased expression patterns were primarily involved in biological processes such as cell–cell/matrix adhesion, cell migration/spreading, embryonic development, and glycolytic process, mainly enriched in the signaling pathways related to cell–cell/matrix interactions (such as tight junction and gap junction, ECM-receptor interaction, and regulation of actin cytoskeleton), reproduction (such as GnRH, estrogen, and insulin signaling pathway), and other testicular development or function (such as VEGF, MAPK, and glycolysis). There are numerous cell adhesion and junctional molecules involved in forming the interactions and communication between germ and Sertoli cells and apposed Sertoli cells and in doing so, facilitate the migration of developing GCs from the basal to the adluminal compartment of the seminiferous epithelium (25, 26). Remarkably, these cell–cell adhesion and junction events are closely related to the underlying actin-based Sertoli cell cytoskeleton, which is being used by various cell junctions for their attachment (25). The actin cytoskeleton is an abundant protein in all eukaryotic cells which plays an integral role in many fundamental processes including cell division, differentiation, motility, and intracellular trafficking (27), as well as their indispensable contribution to testicular development and function (28). SCs have glycolytic activity to provide lactate as an energy source to GCs, being essential for the success of spermatogenesis (29). The estrogen signaling is essential for the normal progression of spermatogenesis (including GC development), and its functional loss can result in male infertility (30). The insulin signaling has been reported to be involved in early development of the testis, proliferation of SCs, and proliferation and differentiation of GCs (31). The VEGF signaling acts directly on male GCs to promote proliferation (32). The MAPK signaling pathway is implicated in the regulation of several testicular cell functions, including proliferation and meiosis of GCs, proliferation and lactate generation of SCs, and cell junction dynamics (33). Collectively, our results imply that these proteins with decreased expression were participated in the regulation of early testicular cell development and microenvironment through a variety of pathways.

Functional annotations indicated that the proteins with consistently increased expression patterns were involved in the regulation of formation of functional spermatozoa at the postspermatogenesis phase (such as cilium assembly, microtubule-based movement, sperm fibrous sheath, and principal piece), in the positive regulation of mitosis, in the negative regulation of GC apoptotic process, and in the actin complex. It is believed that actin-associated protein complexes have important roles in GC movement to traverse the BTB and acrosome formation during spermiogenesis (34). Our KEGG pathway enrichment analysis revealed that these proteins were mainly enriched in the pathways, including reproduction (such as progesterone-mediated oocyte maturation, oocyte meiosis, mTOR, and AMPK signaling pathway), testicular cell functions (such as cell cycle, apoptosis, autophagy, and endocytosis), and cell metabolism, such as glycolysis. Among these, the AMPK signaling pathways have been reported to be implicated in the cell junction dynamics, proliferation of SCs, and lactate supply *via* glycolysis (33). Collectively, our results suggest that these proteins with decreased expression could be participated in the regulation of early testicular cell development and microenvironment through a variety of pathways. On the basis of our data and these best available evidence, we speculate that the pathways that were mediated by these upregulated proteins may be mainly involved in regulating the middle and late stages of spermatogenesis, such as meiotic progression and formation of sperm.

To get a more detailed insight into the significance of the identified proteins and mine the functional candidate genes implicated in various stages of Tibetan sheep spermatogenesis, we constructed the PPI network based on the pathways and functional modules assigned by DAPs. Among these, 23 mitosis-related proteins were used to establish the PPI network by using Cytoscape and three genes in the PPI network were identified as hub genes, including CTNNB1, CUL4B, and RUVBL1. For instance, a recent study revealed that CTNNB1 is an essential regulator of murine spermatogonial stem cell fate to induce its differentiation into spermatogonia (35). In this study, we found that decreases in mRNA and protein levels of CTNNB1 with increasing age, suggestive of its importance for the early stages of Tibetan sheep spermatogenesis. A total of 39 meiosis-related proteins were used to construct the PPI network, in which three genes with the highest edges (CDK1, PPP3CA, and PPP3CB) were considered the core genes of the network. Previous studies demonstrated that CDK1, a known cell cycle regulator, is required for completion of metaphase progression in spermatocytes, and its loss of function can result in meiotic arrest and male infertility (36). Our results indicated that CDK1 is gradually upregulated in developmental testes of Tibetan sheep and hence suggestive of the potential importance for meiotic progression.

A total of 97 spermiogenesis-related proteins were used to create the PPI network; three genes (i.e., DYNC2LI1, KIF3A, and IFT88) were the core of the network. KIF3A, a well-known member of the family of kinesins, is thought to be involved in regulating the formation of spermatozoa-specific flagella, and its depletion causes the severe impairments in sperm tail formation (37). IFT88 is a component of the core intraflagellar transport complex required for the assembly and maintenance of flagella of in mammalian sperm tails (38). Our findings showed that the protein abundance of seven members of the family of kinesins (i.e., KIF2A/C, KIF3A/B, KIF5B, KIF15, and KIF23) and ten members for constituting the intraflagellar transport complex (i.e., IFT20/22/27/43/46/52/80/88/122/172) was significantly increased in the postpubertal and adult Tibetan sheep testes compared with those in prepubertal testes (especially KIF3A/B and IFT20/46, all fold changes > 5.0). Thus, these results alluded that they could play the crucial roles in the biogenesis of peculiar structures for the formation of mature sperm in Tibetan sheep testis.

Normal spermatogenesis requires the disassembly and reassembly of cell junctions between SCs and specific GCs and between adjacent SCs (involving the restructuring of tight junctions, adherens junctions, and gap junctions) on which constitute the BTB and provide the favorable microenvironment for maintaining spermatogenesis, with great repercussions for the development and function of each other (6, 39). In this study, we also constructed an interaction network of proteins involved in the testicular microenvironment based on the annotated biological functions derived from the literature and our functional enrichment analysis. The results showed that a number of integrin proteins (such as ITGA1/5/6 and ITGB1/2) possessed relatively high degree of connectivity with other genes of the network. The integrins are well-known as cell adhesion molecules and exert their effects *via* ECM–receptor interaction (40). In mammalian testis, the self-renewal and differentiation of spermatogonial stem cells occurring at early stages of spermatogenesis are programmed at a unique microenvironment (niche), which is surrounded mainly by SCs, basement membrane, and extracellular matrix, and is strictly modulated by cell adhesion molecules including integrins (such as ITGA4/A6/A7/A9/B1), used for connecting the spermatogonial stem cells to the basement membrane components, as reviewed by Chen et al. (41).

This network also contained the genes enriched in cell adhesion molecules (such as CDH3 and CD34), adherens junction (such as CTNNB1/D1, EGFR, WAS, PTPN6, IQGAP1, and ITGA6), ECM–receptor interaction (such as LAMA1/3/4, NPNT, COL4A6, and DAG1), and gap junction pathways (such as GRB2 and EGFR). These results indicate that changes in the expression of these proteins may be potentially play the shared roles in regulating cellular development, morphogenesis, and BTB integrity that allow the movement of GCs within the seminiferous epithelium and the timely release of spermatids during testis development in Tibetan sheep. Moreover, the constructed network contained 10 glycolysis-related genes; of which, two genes (PGK2 and PGAM2) were found to be synchronously upregulated at both the mRNA and protein levels in postpubertal and adult Tibetan sheep testes. PGK2, an important glycolytic isozyme that catalyzes the first ATP-generating step in the glycolysis pathway, has proven to be of great importance for sperm motility and male fertility in mice (42). Given the acknowledged importance of lactate produced by the SCs through the glycolytic metabolism for developing GCs (29), we speculated that these identified glycolysis-related proteins may be involved in the regulation of lactate production of SCs *via* changes in expression to provide a steady stream of energy substrates for various GCs in developmental testes of Tibetan sheep.

## Conclusion

This is the first study describing a high-throughput global protein changes during testicular development in sheep. Our results indicated that numerous protein-coding genes were differentially expressed in a development-dependent manner in Tibetan sheep testes where variations in the levels of these proteins did not always synchronize with variations in their transcript levels. These findings may imply that the differences in abundance of these proteins mainly contribute to the testicular cell development and their surrounding microenvironment remodeling at various stages of spermatogenesis *via* divergent signaling pathways. This study provide fundamental insights into how protein function during spermatogenesis of Tibetan sheep, as well as offer abundant candidate genes and pathways for future in-depth studies regarding the regulatory mechanisms underlying testicular development and spermatogenesis in postnatal sheep.

## Data Availability Statement

The datasets presented in this study can be found in online repositories. The names of the repository/repositories and accession number(s) can be found in the article/Supplementary Material.

## Ethics Statement

The animal study was reviewed and approved by Laboratory Animal Welfare and Ethics Committee of Gansu Agricultural University.

## Author Contributions

TL conceived and designed the study and wrote the original draft. MS, HS, and HC collected and prepared samples. TL and HW performed the experiments. TL, RL, and XA analyzed and visualized the formal data. QL conducted the literature search and screening. YM supervised the study. YZ and YM contributed to revisions of the manuscript. All authors read and approved the manuscript.

## Funding

This study was financially supported by the Youth Fund of College of Animal Science and Technology of Gansu Agricultural University (GAU-DK-QNJJ-202104).

## Conflict of Interest

The authors declare that the research was conducted in the absence of any commercial or financial relationships that could be construed as a potential conflictof interest.

## Publisher's Note

All claims expressed in this article are solely those of the authors and do not necessarily represent those of their affiliated organizations, or those of the publisher, the editors and the reviewers. Any product that may be evaluated in this article, or claim that may be made by its manufacturer, is not guaranteed or endorsed by the publisher.
