# Supplementary material for: Proteome Informatics in Tibetan Sheep (Ovis aries) Testes Suggest the Crucial Proteins Related to Development and Functionality
Source: Front Vet Sci. 2022 Jul 15;9:923789. doi: 10.3389/fvets.2022.923789 (PMC9334778; doi:10.3389/fvets.2022.923789)
Supplement: Supplementary file 1 [file Table_1.DOCX]

**Table S1.** Primer pairs of mRNAs used for qPCR.

| Gene name | Primer sequence-Forward (5′-3′) | Primer sequence-Reverse (5′-3′) | Amplicon (bp) |
| --- | --- | --- | --- |
| ARPC5 | GCGGCAAGGAAACATGACAG | AAGACTCGAACGATGGACCC | 297 |
| ASF1B | AGAATCCGAGCCCTTTCCAC | TCTTCCACTCCAGGTCGTCT | 87 |
| CLIC4 | ATTGTCAAGGTGGTGGCGAA | ACGTTTCTCTCGCAAGTGCT | 197 |
| CTNNB1 | GGGTCCTCTGTGAACTTGCT | GGCTTGACCTCAGACATTC | 154 |
| DLD | GCTGGAGTCGTGTGTACTGT | CATTCAAGCATGTCCCACCG | 240 |
| GPX4 | TGTGGTTTACGGATCCTGGC | CCTTGGGCTGGACTTTCAT | 182 |
| HSPA2 | ATCTGCTGCTACTCGACGTG | TCCTTTTGATGAGCGGGGTC | 81 |
| LMNA | ACGAGTGGATGCTGAGAACC | TTCCTCTCCGCTGACTGTCT | 276 |
| ODF2 | AAGGACCGCTCTTCAACTCC | AGGCAGTGAGGTGGATTCTTG | 192 |
| PGAM2 | CCACTATGTCTACCCACCGC | TCTGCATCGAACCAACCACA | 91 |
| PGK2 | CAGAAGTGGAGAAGGCGTGT | CCTAGCTTGGAGAGGGAT | 171 |
| PIN1 | CAGGCCGGGTGTACTACTTC | CCCCTTGTCCATTTTTGCCG | 96 |
| RBMXL2 | CCCAAGGACTAGTGCAAGGAA | AAGACGACTAGTAAGCAGGTTCA | 197 |
| RDH11 | ACTGACCCTGATTCATGGAGG | GCACTAGGGTCCAGTTTA | 181 |
| ROPN1L | TTGCACAAGCAGTGTAGCCA | ATCGCAGTGTTCAGTGACCC | 191 |
| SEC23IP | CCCCCGTTCAGATGTACCAG | GTAGAAAAGGAGGCGGTGCT | 144 |
| TDRD6 | GCAAAGAAACACACGGCGTA | CTCGTTGGATCGCCTGGATA | 90 |
| ZPBP | GGAAGTTTTGGGCAGACGTG | CCAGGGCTGCAGATTACACA | 156 |
| β-actin | TATTGCTGCGCTCGTGGTTG | GTCAGGATGCCTCTCTTGCT | 186 |
